# Supplementary material for: Empirical methods for controlling false positives and estimating confidence in ChIP-Seq peaks
Source: BMC Bioinformatics. 2008 Dec 5;9:523. doi: 10.1186/1471-2105-9-523 (PMC2628906; doi:10.1186/1471-2105-9-523)
Supplement: Additional File 1 — A variety of html documents from the USeq web site detailing the available applications, their best usage, output file type descriptions, command line menus, etc. [file 1471-2105-9-523-S1.zip › USeqUserGuides/JavaDoc.zip!/JavaDoc/trans/main/IntervalGraphPrinter.html]

IntervalGraphPrinter


|  |  |  |
| --- | --- | --- |
| **Overview** \| **Package** \| **Class** \| **Use** \| **Tree** \| **Deprecated** \| **Index** \| **Help** | |  |
| **PREV CLASS**   **NEXT CLASS** | **FRAMES**    **NO FRAMES**     **All Classes** |  |
| SUMMARY: NESTED \| FIELD \| CONSTR \| METHOD | DETAIL: FIELD \| CONSTR \| METHOD |  |


---


## trans.main Class IntervalGraphPrinter

```
java.lang.Object
  trans.main.IntervalGraphPrinter
```

---

``` public class IntervalGraphPrinter extends java.lang.Object ```

Prints an array of Interval as an '.sgr' file for import into IGB.

---

**Constructor Summary**

`IntervalGraphPrinter(java.lang.String[] args)`


| **Method Summary** | |
| --- | --- |
| `java.lang.String` | `convertToBED(Interval[] intervals)`             Converts an array of Interval to a .bed String format. |
| `java.lang.String` | `convertToSGR(Interval[] intervals)`             Converts an array of Interval to a .sgr String format. |
| `static void` | `main(java.lang.String[] args)` |
| `static void` | `printDocs()` |
| `void` | `printSaveIntervalScoreGraphFiles()`             Prints and saves a Affy .sgr file |
| `static void` | `printScores(Interval[] intervals, java.io.File file, boolean printRatios)`             Prints to a File a line for every Oligo in each Interval. |
| `void` | `processArgs(java.lang.String[] args)`             This method will process each argument and assign any new varibles |
| `void` | `sortIntervals()`             Sorts Interval[] based on subWindow if present, if not, then uses best window |

**Methods inherited from class java.lang.Object**

`equals, getClass, hashCode, notify, notifyAll, toString, wait, wait, wait`

**Constructor Detail**

### IntervalGraphPrinter

```
public IntervalGraphPrinter(java.lang.String[] args)
```


**Method Detail**

### processArgs

```
public void processArgs(java.lang.String[] args)
```

:   This method will process each argument and assign any new varibles

---


### printSaveIntervalScoreGraphFiles

```
public void printSaveIntervalScoreGraphFiles()
```

:   Prints and saves a Affy .sgr file

---


### printScores

```
public static void printScores(Interval[] intervals,
                               java.io.File file,
                               boolean printRatios)
```

:   Prints to a File a line for every Oligo in each Interval.
    Tab delimited: Chromosome, Start of the oligo, the log2(aveT/ aveC ratio).

---


### convertToSGR

```
public java.lang.String convertToSGR(Interval[] intervals)
```

:   Converts an array of Interval to a .sgr String format.

---


### convertToBED

```
public java.lang.String convertToBED(Interval[] intervals)
```

:   Converts an array of Interval to a .bed String format.

---


### sortIntervals

```
public void sortIntervals()
```

:   Sorts Interval[] based on subWindow if present, if not, then uses best window

---


### main

```
public static void main(java.lang.String[] args)
```

---


### printDocs

```
public static void printDocs()
```


---


|  |  |  |
| --- | --- | --- |
| **Overview** \| **Package** \| **Class** \| **Use** \| **Tree** \| **Deprecated** \| **Index** \| **Help** | |  |
| **PREV CLASS**   **NEXT CLASS** | **FRAMES**    **NO FRAMES**     **All Classes** |  |
| SUMMARY: NESTED \| FIELD \| CONSTR \| METHOD | DETAIL: FIELD \| CONSTR \| METHOD |  |


---
